# Recombinant TSH Stimulated Remnant Ablation Therapy in Thyroid Cancer: The Success Rate Depends on the Definition of Ablation Success—An Observational Study

**DOI:** 10.1371/journal.pone.0120184

**Published:** 2015-03-20

**Authors:** Anouk N. A. van der Horst-Schrivers, Wim J. Sluiter, Anneke C. Muller Kobold, Bruce H. R. Wolffenbuttel, John T. M. Plukker, Peter H. Bisschop, John M. de Klerk, Imad Al Younis, Paul Lips, Jan W. Smit, Adrienne H. Brouwers, Thera P. Links

**Affiliations:** 1 Department of Endocrinology, University of Groningen, University Medical Center Groningen, Groningen, the Netherlands; 2 Department of Laboratory Medicine, University of Groningen, University Medical Center Groningen, Groningen, the Netherlands; 3 Department of Surgery, University of Groningen, University Medical Center Groningen, Groningen, the Netherlands; 4 Academic Medical Center, University of Amsterdam, Department of Endocrinology, Amsterdam, the Netherlands; 5 Meander Medical Center Amersfoort, Department of Nuclear Medicine, Amersfoort, the Netherlands; 6 Leiden University Medical Center, Department of Nuclear Medicine and Molecular Imaging, Leiden, the Netherlands; 7 VU University Medical Center, Department of Internal Medicine/Endocrinology, Amsterdam, the Netherlands; 8 Radboud University Nijmegen Medical Center, Department of Internal Medicine, Nijmegen, the Netherlands; 9 Department of Nuclear Medicine and Molecular Imaging, University of Groningen, University Medical Center Groningen, Groningen, the Netherlands; Uppsala University, SWEDEN

## Abstract

**Introduction:**

Patients with differentiated thyroid cancer (DTC) are treated with (near)-total thyroidectomy followed by remnant ablation. Optimal radioiodine-131 (^131^I) uptake is achieved by withholding thyroid hormone (THW), pretreatment with recombinant human Thyrotropin Stimulating Hormone (rhTSH) is an alternative. Six randomized trials have been published comparing THW and rhTSH, however comparison is difficult because an uniform definition of ablation success is lacking. Using a strict definition, we performed an observational study aiming to determine the efficacy of rhTSH as preparation for remnant ablation.

**Patients and Methods:**

Adult DTC patients with, tumor stage T1b to T3, Nx, N0 and N1, M0 were included in a prospective multicenter observational study with a fully sequential design, using a stopping rule. All patients received remnant ablation with ^131^I using rhTSH. Ablation success was defined as no visible uptake in the original thyroid bed on a rhTSH stimulated 150 MBq ^131^I whole body scan (WBS) 9 months after remnant ablation, or no visible uptake in the original thyroid bed on a post therapeutic WBS when a second high dose was necessary.

**Results:**

After interim analysis of the first 8 patients, the failure rate was estimated to be 69% (90% confidence interval (CI) 20-86%) and the inclusion of new patients had to be stopped. Final analysis resulted in an ablation success in 11 out of 17 patients (65%, 95% CI 38-86%).

**Conclusion:**

According to this study, the efficacy of rhTSH in the preparation of ^131^I ablation therapy is inferior, when using a strict definition of ablation success. The current lack of agreement as to the definition of successful remnant ablation, makes comparison between different ablation strategies difficult. Our results point to the need for an international consensus on the definition of ablation success, not only in routine patient’s care but also for scientific reasons.

**Trial Registration:**

Dutch Trial Registration NTR2395

## Introduction

Patients with differentiated thyroid cancer (DTC) are initially treated with (near)-total thyroidectomy followed in most cases by radioiodine-131 (^131^I) therapy (‘remnant ablation’) [1,2]. The aim of remnant ablation is to eliminate normal remnant thyroid tissue and to destroy radioiodine-avid tumor cells; thus eliminating benign sources of thyroglobulin (Tg) and lowering the rate of tumor recurrence. Optimal ^131^I uptake is achieved by withholding thyroid hormone therapy (THW), a practice that induces hypothyroidism, leading to a diminished rate of renal clearance of administered ^131^I and a prolonged Thyrotropin Stimulating Hormone (TSH) stimulation [3]. With the introduction of recombinant human (rh)TSH, Thyrogen, Genzyme Therapeutics), remnant ablation after pretreatment with rhTSH during euthyroidism has been proposed as an alternative to THW. Using rhTSH avoids development of symptoms resulting from hypothyroidism, maintains the quality of life and achieves a lower whole body radiation as compared to preparation with THW [4,5].

Six randomized trials have been published comparing TWH and rhTSH stimulated remnant ablation, with different success rates ranging between 70 and 94% (Table 1) [5–10]. Several of these studies, however, were underpowered because the primary endpoint was not ablation success. The large difference in outcome between these studies can be explained by several factors, such as the inclusion of patients from different risk categories, differences in the extent of the primary surgery and the use of different definitions of ablation success [11].

**Table 1:** Performed randomized controlled trials comparing ablation success rates between recombinant human (rh)TSH and thyroid hormone withdrawal.

Author	Tumor stages	N	^131^I dose	Definition of ablation success	Evaluation using	Success rates (%)						Remarks
						Based on WBS		Based on Tg		Based on WBS (ultrasound) & Tg		
						rhTSH group	THW group	rhTSH group	THW group	rhTSH group	THW group	
Lee [6]	T1, T2, T3, N1a	291	1.1 GBq	No visible uptake or < 0.1% on WBS & normal ultrasound and Tg < 1.0 ng/mL	THW	91.3	94.4	92.8	91.0			3 groups; T4 THW, T3 THW & rhTSH (T4 stopped for 4 days)
Schlumberger [7]	T1/any N & T2N0	729	1.1 GBq or 3.7 GBq	Normal ultrasound & Tg < 1 ng/mL, in case of TgAb no visible uptake or ≤ 0.5% on WBS	rhTSH	**	**	94.9	95.6	91.9	92.8	Tg < 1.0 ng/mL (without TgAb) at time of ablation in 315 patients
Mallick [8]	T1-T3/any N	438	1.1 GBq or 3.7 GBq	No visible uptake or < 0.1% on WBS and/ or Tg < 2.0 ng/mL	rhTSH	93.8	93.8	87.6	86.2	87.1	86.7	Tg < 2.0 ng/mL (without TgAb) at time of ablation in 110 patients
Pacini [9]	T2-T4/any N, T0-T1/N1*	63	3.7 GBq	No visible uptake or < 0.1% on WBS	rhTSH	100 75^#^	100 85.7^#^	83.3	85.7			
Taieb [5]	T1-T3/any N	74	3.7 GBq	Not predefined Tg cut-off level < 0.8 ng/mL	rhTSH	94	100	91.7	97.1	88.9 69.5^#^	97.1 88.6^#^	
Chianelli [10]	T1 (> 1 cm)/N0	24	2.0 GBq	Not predefined (no visible uptake on WBS)	THW	90.5	95.2	85.0	90.0	75.0	90.0	

* minimally invasive in case of T4

** WBS performed only in 23 patients

# Based on no visible uptake in the original thyroid bed on the WBS

WBS: Whole body scan, Tg: Thyroglobulin, THW: thyroid hormone withdrawal, rhTSH: recombinant human TSH.

Due to conflicting data regarding the possible benefits and drawbacks and the efficacy of rhTSH stimulation on ablation success, we performed an observational study aiming to confirm the efficacy of the use of rhTSH as preparation for remnant ablation. We defined successful ablation as no visible uptake on a subsequent whole body scan (WBS) [12]. The secondary aim was to establish ablation success based on the additional Tg determination. We used an observational study design using continuous sequential analysis, as described earlier and for safety reasons we defined stopping rules of the occurrence of ablation failure [13].

## Patients and Methods

### Aim of the study

This was a prospective multicenter observational study with a fully sequential design (see statistical analysis). The objective was to evaluate the success rate of ^131^I ablation therapy using rhTSH stimulation. We considered a failure rate of 10% acceptable as compared to the failure previously described in DTC patients after remnant ablation using THW [14–16].

The protocol for this trial and supporting CONSORT checklist are available as supporting information; see S1 CONSORT Checklist and S1 Protocol.

### In and exclusion criteria

Adult patients (18 years or older), who were diagnosed with histological proven DTC, low and high risk (according the American Joint Committee on Cancer, 7^th^ edition), with a TNM stage of T1b (larger than 1cm), T2, T3, N0, N1 and M0 were eligible for inclusion. Exclusion criteria were stage T4 disease and M1, known before ablation, as well as pregnancy. Since rhTSH can lead to more severe side effects in patients with end stage renal disease we excluded patients with an abnormal renal function defined by a serum creatinine > 130 μmol/L or a creatinine clearance rate of less than 40 mL/min. Patients who had a major concurrent medical disease, a previous history of a malignancy or had received an investigation with iodine-containing contrast agents within four months prior to the ^131^I ablation treatment were excluded. After December 2010 the inclusion of patients was permitted if the concurrent disease or prior malignancy did not reduce survival expectation to less than one year. In case of recent (< four months) exposure to iodine containing contrast agents the remnant ablation was postponed for four months.

All patients provided informed written consent. The study was approved by the Medical Ethics Review committee of all participating centers (University of Groningen, University Medical Center Groningen (UMCG), Academic Medical Center (AMC), University of Amsterdam, Meander Medical Center Amersfoort, Leiden University Medical Center, VU University Medical Center). This trial was registered in the Dutch Trial Registration (number: NTR2395), at the 29^th^ of June 2010, due to logistic problems the first patient was already include 6 days prior to this registration. The authors confirm that all ongoing and related trials for this drug are registered.

### Study design

After (near)-total thyroidectomy and histological confirmation of DTC, thyroid hormone treatment (Levothyroxine) was started at a dosage of 2 μg/kg bodyweight/day to reach a TSH level < 0.3 mU/L. Three to six weeks after thyroidectomy, an ultrasound of the neck was performed to confirm the absence of significant thyroid remnant tissue. Six weeks after surgery 0.9 mg rhTSH was administered by intramuscular injection on 2 consecutive days; 24 hours later blood was withdrawn for measurement of Tg and Tg antibodies (Ab) and 3.7 GBq (100 mCi) ^131^I was administered. Patients underwent a ^131^I post therapeutic WBS 7 days later.

Six months after the remnant ablation the neck ultrasound was repeated. When suspicious lymph nodes were encountered a fine needle aspiration (FNA) was performed. In case of a positive FNA, re-surgery was considered and patients were excluded from the analysis. Nine months after the remnant ablation 0.9 mg rhTSH was administered intramuscularly on 2 consecutive days; one day later patients received 150 MBq (4 mCi) ^131^I. Serum TSH, Tg and TgAb were measured, and a WBS was performed 3 days after the last rhTSH administration.

Patients with TgAb, and/or patients with a Tg < 1.0 ng/mL at the time of the remnant ablation (despite visible uptake in the original thyroid bed on the post therapeutic WBS) were considered to have “unreliable” Tg results: undetectable Tg without measurable TgAb. These patients, as well as those suspected of having distant metastases (visualized on the initial post therapeutic WBS), received a second treatment, now with 5.55 GBq ^131^I after THW, 6 months after the initial remnant ablation. [12,17,18]. All patient were on a low iodine diet for 1 week prior to all ^131^I treatments.

In the individual participating centers all scans and laboratory results were evaluated in order to make decisions as to treatment.

The primary and secondary endpoints were re-evaluated at the principal study center, the UMCG. All WBS scans were reviewed by two independent nuclear physicians (AHB and BvE) who were unaware of the outcome of the patients. There were no differences in their readings.

### Definition of ablation success

The primary definition of ablation success was: having no visible uptake in the original thyroid bed on the rhTSH stimulated 150 MBq ^131^I WBS, or no visible uptake in the original thyroid bed on the post therapeutic WBS when a second dose of ^131^I was administered. The second definition of ablation success was: absence of visible uptake in the original thyroid bed combined with a serum stimulated Tg level of < 1 ng/mL, when the Tg measurement was judged to be reliable.

### Thyroglobulin assay

Tg measurements were performed in the University Medical Center Groningen using a commercial immunoradiometric assay (ThermoFischer, formerly Brahms Tg-Plus, Heningsdorf, Germany). The Brahms Tg-Plus assay is calibrated against the CRM 457 standard. This assay has an analytical sensitivity of 0.1 ng/mL and a functional sensitivity of 0.3 ng/mL (according to Clinical and Laboratory Standards Institute (CLSI) EP5 guideline). The TgAb were also measured using a commercial radioimmuno assay (Brahms anti-Tg assay, Heningsdorf, Germany); levels exceeding 46 U/mL were considered positive for the presence of Ab, reference value verification according to CLSI C28-A3 guideline.

### Statistical analysis

#### Interim evaluation and stopping rules

This prospective study had a sequential design with a stopping rule equipped with two boundaries, as described earlier by van der Zee et al [13]. This analysis makes it possible to use every single consecutive remnant ablation in an interim analysis. An ablation failure of 10% was considered to be acceptable based on the reported literature [14–16].

Upper and lower boundaries were established on a true failure rate of 10% and its alternative of 20%. The study was to be terminated after the crossing of either boundary. After passing the upper boundary preparation with rhTSH would be concluded to be less effective than preparation using THW. After passing the lower boundary the failure rate of rhTSH stimulation would be considered to be below 20% and thus acceptable. So, the preset upper boundary would allow premature stopping in case of an observed failure rate in excess of the acceptable rate of 10%. We calculated that a maximum total of 144 patients were needed (constant α = 0.014, cumulative α = 0.05; β = 0.10). This implied that with a true failure rate of 10%, the probability of passing the upper boundary would be below 5%. A preset lower boundary was made (constant α = 0.2638; cumulative α = 0.05), allowing premature stopping of monitoring. Passage of that boundary would mean testing for futility, because with a true failure rate of 20%, the probability of first passing this lower boundary, with subsequent passage of the upper boundary, would be below 5%.

In case of passing the upper boundary, it was agreed in accordance with the Medical Ethics Review committee that the study should be stopped prematurely because the use of rhTSH in remnant ablation would be inferior in terms of ablation success.

## Results

### Patients

**Fig 1:**
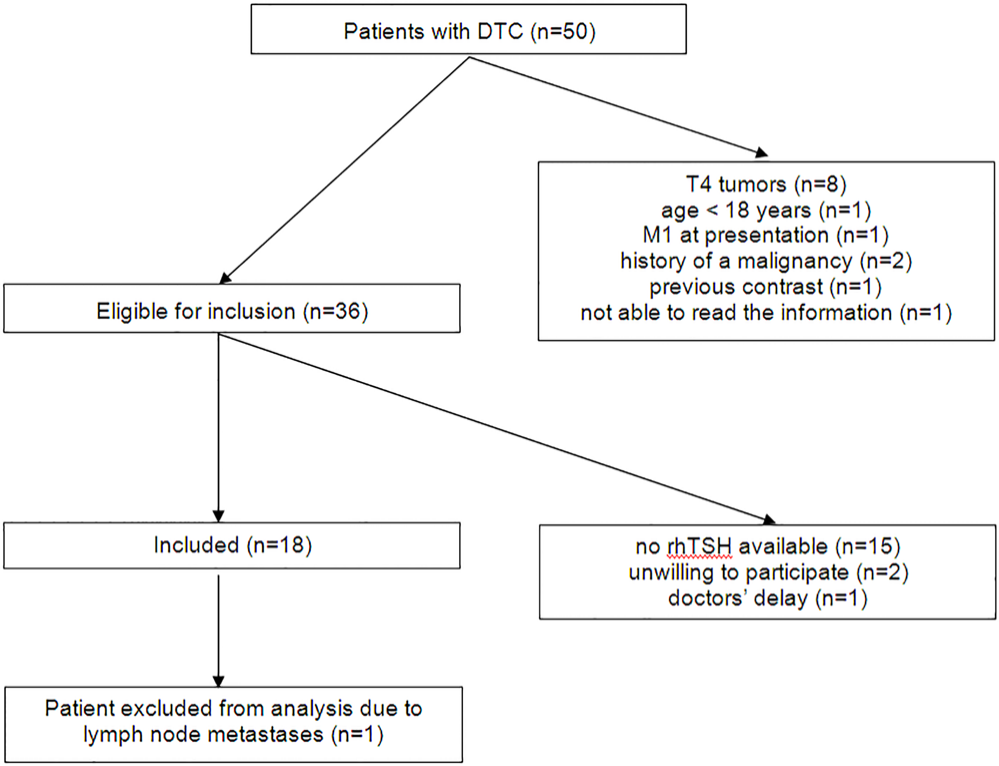
Flow chart of the inclusion of patients with differentiated thyroid carcinoma (DTC).

Between July 2010 and June 2011 a total of 50 patients were referred for remnant ablation after (near)-total thyroidectomy because of a DTC in two (UMCG 48 patients, AMC 2 patients) of the five participating centers. Eighteen patients (36.0%) were included in the study (Fig. 1, Table 2). As rhTSH was not available in the Netherlands between February 2011 and December 2011 due to restricted supply, the study was not started in the other centers although approval of the local Medical Ethic Review committees had been obtained. All patients except one (number 13) had no evidence of significant thyroid remnant tissue on the ultrasound, performed three to six weeks after thyroidectomy. In patient number 13 there was a questionable small remnant (< 9 mm). One patient with moderately reduced kidney function was included (number 7), one patient was excluded after 6 months from the final analysis as he was demonstrated to have lymph node metastases and subsequently underwent an operation. The remaining patients did not have any suspected lymph nodes on the ultrasound. Urinary iodine concentration were < 100 μg/L, in 12 out of 15 patients (80.0%), with a median of 64 μg/L (range < 39 μg/L—144 μg/L).

**Table 2:** Baseline characteristics and thyroglobulin (Tg) levels at the time of the ablation, and results of evaluation of ablation success.

Characteristics at the time of ^131^I ablation therapy					Results of Tg & WBS, 6 or 9 months after remnant ablation, rhTSH stimulated (1) or after a second high dose after THW (2)	
N^0^	Age	Gender	Tumor	Tg (ng/mL)	Tg (ng/mL)	Visible uptake on WBS
1	46	F	PTC, T2N0	< 0.11	< 0.11	No (2)
2	65	F	PTC, T3N0	< 0.11	0.13	Yes (2)^#^
3	71	M	PTC, T2N0^a^	0.38	0.15	Yes (2)^#^
4	58	F	PTC, T3N0	1.7	16	Yes (2)^#^
5	59	M	PTC, T3N0	9.4	9.5	No (1)
6	52	M	FTC, T3N0	6.3	< 0.11	No (1)
7	56	F	PTC, T1aN0^a^	1.8	0.32	No (1)
8	43	M	PTC, T3N0^a^	4.8	< 0.11	No (1)
9	29	F	PTC, T2N0^a^	0.83	< 0.11	Yes (2)^#^
10	58	F	FTC, T2N0	< 0.11*	< 0.11	Yes (2)
11	26	F	PTC, T2N0	< 0.11	< 0.11	No (2)
12	54	M	PTC, T1bN0^a^	< 0.11	< 0.11	No (2)
13	46	F	FTC, T2N0^b^	3.6	< 0.11	No (1)
14	39	F	PTC, T2N0^a^	8.7	11.0	No (1)
15	34	F	PTC, T1bN0	0.14*	< 0.11	Yes (2)
16	24	F	PTC,T2N0	0.24	< 0.11	No (2)
17	77	F	PTC, T1N0	3.0**	< 0.11	No (1)

*TgAb positive,

**Measured at the Academic Medical Center (chemiluminescence immunoassay (LUMI-test Tg, BRAHMS, Berlin, Germany). Detection limit: 1 pmol/L. Total assay variation: 7–13%).

^a^multifocality,

^b^including foci of PTC

F: female, M: male, PTC: papillary thyroid cancer, FTC: follicular thyroid cancer, Tg: Thyroglobulin, Ab: antibodies, WBS: whole body scan

^#^Patients 2,3,4 and 9 were not succesfully ablated in the interim analysis

### Interim analysis

In August 2011, the first 8 patients (numbers 1, 2, 3, 4, 5, 6, 7 and 9) were included in the planned interim analysis which evaluated ablation success nine months after inclusion. In 4 patients (numbers 2, 3, 4 and 9) ablation was not successful, hence the upper boundary of the stopping rule was passed. Accounting for the sequence of the events, the failure rate was estimated to be 69% (90% confidence interval (CI) 20–86%). As a result remnant ablation using rhTSH was concluded to be inferior to remnant ablation after THW. According to the agreement with the Medical Ethics Review committee the inclusion of new patients had to be stopped immediately.

### Final analysis—ablation success

Ten out of 17 patients received a second high dose, 7 patients because of undetectable Tg without measurable TgAb, 2 patients (numbers 10 and 15) had TgAb at the time of ablation, 103 U/mL and 104 U/mL respectively. One patient (number 4) received a second high dose because of clinical suspicion of multiple pulmonary metastases. Computed Tomography (CT) imaging performed 1 month after the remnant ablation could not, however, verify these metastases and the post therapeutic WBS after the second high dose also did not show these possible metastases. Using the definition of no visible uptake on rhTSH stimulated 150 MBq ^131^I WBS or no visible uptake in the original thyroid bed on a ^131^I post therapeutic WBS, the result was ablation success in 11 out of 17 patients (65%, 95%CI 38–86%) (Table 2). Using the stricter definition of successful ablation (no visible uptake in the original thyroid bed and a stimulated Tg level of < 1 ng/mL, if reliable), the result was a successful ablation in 9 out of 17 patients (53%, 95% CI 28–78%).

## Discussion

This study was performed to confirm the efficacy of ^131^I ablation therapy using rhTSH. Surprisingly, our study had to be stopped as the evaluation of the first 8 patients demonstrated that rhTSH stimulated remnant ablation with 3.7 GBq ^131^I was inferior to historical success rates of remnant ablation using THW. Two recently published large randomized trials [7,8] found no differences between THW and rhTSH, and a much higher ablation success rate was observed.

It must be stressed that our observational study design with stopping rule differed from the two randomized studies that used either a non-inferiority design [8] or an equivalence framework [7]. However, other factors also contribute to the difference in outcome and these should be discussed more in depth in order to expand the discussion on the indication of the use of rhTSH.

In general, evaluation of ablation success is based on a surrogate endpoint instead of the hard clinical endpoint of recurrence or mortality [19,20]. As shown in Table 1, the definition of this surrogate endpoint differs among the published studies, and is also not consistent with current guidelines [21–23]. There is no consensus regarding preference for THW or rhTSH during the evaluation of ablative therapy, the cut-off value of Tg [24], the definition of a “negative” WBS (no visible uptake or uptake < 0.5%) and the approach to patients with an undetectable Tg without measurable TgAb at the time of ablation. Using a more strict definition; i.e THW stimulated Tg < 1.0 ng/mL, no visible uptake on a WBS and a negative neck ultrasound, used in the present study, will result in a lower ablation success rate. This is also illustrated in the studies by Maenpaa et al. and Pacini et al, in which comparably low success rates of 52% and 54% were reported [25,26]. Using an even more strict cut-off level, feasible with the current Tg assays, would have even led to lower success rate in our study. A stricter definition of ablation success could bring to light the true difference between THW and rhTSH preparation.

It is unclear from the literature whether the finding of undetectable Tg in the absence of TgAb is a proof of complete thyroidectomy, or is due to “unreliable” Tg and TgAb assays. According to our strict ablation protocol we prescribed a second high dose of ^131^I in the 7 patients with an undetectable Tg without measurable TgAb at the time of ablation. We assumed that the absence of Tg did not reflect a true complete thyroidectomy, but rather insufficient and “unreliable” Tg and TgAb assays. The fact that a remnant could be visualized in three out of seven patients partly confirms this assumption. This is in accordance with earlier results from our group, in which we showed uptake in the original thyroid bed after THW in 95% of 94 patients, indicating the presence of remnant thyroid tissue despite undetectable Tg without TgAb [27]. However some other explanations are possible. First, when surgery is performed by only a restricted number of experienced surgeons, then an undetectable Tg may correlate better with true total thyroidectomy, as might also be the case in other studies [6–8]. In our study the 18 patients underwent surgery in nine different hospitals, which is still a common situation in the Netherlands and the majority of other countries. Second, the undetectable Tg without measurable TgAb in the 4 remaining patients could also be the result of obtaining the Tg level one day after the last rhTSH injection instead of three days, since previously it was shown that maximum serum Tg levels are obtained three days after the final rhTSH injection [28]. Finally in rare cases thyroid cancer cells express sodium/iodide symporter but are not able to produce Tg [29].

It has to be realized that the reduction of the recurrence rate after remnant ablation is probably the effect of the destruction of iodine sensitive tumor cells, and not of the normal remnant, which is the subject of the definition of ablation success. Normal remnant thyroid tissue may differ from that of tumor cells in that the latter have a reduced ability to take up iodine [30]. So, one can hypothesize that tumor cells require a longer period of TSH stimulation of the sodium-iodide symporter than normal thyroid cells. This suggests that THW can be expected to be more effective for an adequate uptake of iodine by tumor cells. Because no prospective studies are available comparing the effect of TWH and rhTSH stimulated ablation therapy with hard clinical outcomes of recurrence or mortality, the long term outcome of the use of rhTSH has not been proven to be safe on long term outcome. Still, in patients with low risk DTC the disease free and overall survival rate is almost 100%. So the outcome in those low risk patient groups is probably independent of the preablative strategy, and maybe even totally independent of the ablation therapy [2,31–34].

In conclusion, according to our study the efficacy of rhTSH in the preparation of ^131^I ablation therapy is inferior if one uses a strict definition of ablation success and in daily practice. Our data contribute to the debate on the role of rhTSH in the treatment of thyroid cancer patients. The lack of agreement as to the definition of successful remnant ablation must be added to the list of variations in the management of thyroid cancer published recently by Haymart et al. It is of utmost importance to agree on the definition of this endpoint, not only for the sake of routine patient care but also for scientific reasons [35]. Efforts must be made to reach an international consensus as to the definition of ablation success in order to make comparison between different ablation strategies possible.

## Supporting Information

S1 CONSORT Checklist CONSORT Checklist. (DOC)

S1 Protocol I-131 Remnant Ablation in Differentiated Thyroid Cancer-optimal treatment with maximal outcome. (DOC)
