# Supplementary material for: Recombinant TSH Stimulated Remnant Ablation Therapy in Thyroid Cancer: The Success Rate Depends on the Definition of Ablation Success—An Observational Study
Source: PLoS One. 2015 Mar 20;10(3):e0120184. doi: 10.1371/journal.pone.0120184 (PMC4367989; doi:10.1371/journal.pone.0120184)
Supplement: S1 Protocol — (DOC) [file pone.0120184.s002.doc]

**I-131 Remnant Ablation in**

**Differentiated Thyroid Cancer**

*-optimal treatment with maximal outcome-*

**University Medical Center Groningen**

Dr. A.H. Brouwers ^1^

Dr. T.T.H. Phan ^1^

Dr. W.J. Sluiter ^2^

Dr. A.N.A. van der Horst-Schrivers ^2^

Dr. A. Muller Kobold ^3^

Prof. dr. T.P. Links ^2^

**Leiden University Medical Center**

Dr. I. al Younis^1^

Drs. M.B. vd Hiel ^1^

Prof. dr. J.W.A. Smit ^2^

**Academic Medical Center Amsterdam**

Dr. P.H.L.T. Bisschop ^2^

Prof. dr. E. Fliers ^2^

Prof. dr. B. van Eck^1^

**Meander Medical Center Amersfoort**

Dr. J.M.H. de Klerk ^2^

**VU Medisch Centrum**

Prof. dr. P Lips ^2^

*Departments of ^1^ Nuclear Medicine and Molecular Imaging, ^2^ Endocrinology and ^3^Laboratory/Chemistry*

**PROTOCOL TITLE** ‘131I-remnant ablation in differentiated thyroid cancer’

| **Protocol ID** | **Links131I-ablation** |
| --- | --- |
| **Short title** | **131-I remnant ablation in DTC** |
| **Version** | **04** |
| **Date** | **July 2011** |
| **Coordinating investigator/project leader** | ***Prof. dr. T.P. Links, internist-endocrinologist UMCG*** |
| **Principal investigator(s)**  **(in Dutch: hoofdonderzoeker/uitvoerder)** | ***Prof. dr. T.P. Links, internist-endocrinologist UMCG***  ***Mrs. Dr. A.H. Brouwers, nuclear medicine UMCG*** |
| **Sponsor (in Dutch: verrichter/opdrachtgever)** | ***Prof. Dr. T.P. Links, internist-endocrinologist UMCG*** |
| **Independent physician(s)** | ***Dr. J. Pruim, nuclear medicine UMCG*** |
| **Laboratory sites** | ***Dr. A. Muller Kobold, clinical chemist*** |
| **Pharmacy** |  |

**PROTOCOL SIGNATURE SHEET**

| **Name** | **Signature** | **Date** |
| --- | --- | --- |
| **For non-commercial research** |  |  |
| ***Prof.dr. R.A. Dierckx, head of Department NGMB***  ***Prof.dr B.H.R. Wolffenbuttel, Head of Endocrinology Department*** |  |  |
| **Coordinating Investigator:**  ***Prof. dr. T.P. Links, internist-endocrinologist*** |  |  |

**TABLE OF CONTENTS**

INTRODUCTION AND RATIONALE 07

OBJECTIVES 07

STUDY DESIGN 08

STUDY POPULATION 08

Population (base) 08

Inclusion criteria 08

Exclusion criteria 08

Sample size calculation 08

METHODS 09

Main study parameter/endpoint 09

Study procedures 09

Withdrawal of individual subjects 10

Replacement of individual subjects after withdrawal 11 Premature termination of the study 11

SAFETY REPORTING 11

Section 10 WMO event 11

Adverse and serious adverse events 11

Annual safety report 11

STATISTICAL ANALYSIS [12](#__RefHeading___Toc102891176)

Descriptive statistics [12](#__RefHeading___Toc102891177)

Power analysis 12

ETHICAL CONSIDERATIONS 15

Regulation statement 15

Recruitment and consent 15

Benefits and risks assessment, group relatedness 15

Compensation for injury 16

Incentives (if applicable) 16

ADMINISTRATIVE ASPECTS AND PUBLICATION 16

Handling and storage of data and documents 16

Amendments 16

Annual progress report 16

End of study report 16

Public disclosure and publication policy 17

REFERENCES 18

**LIST OF ABBREVIATIONS AND RELEVANT DEFINITIONS**

| **WBS** | **Whole body scan** |
| --- | --- |
| **Dx** | **Diagnostic** |
| **ICRP** | **International Commission on Radiological Protection** |
| **GBq** | **GigaBecquerel** |
| **RIVM** | **Rijksinstituut voor Volksgezondheid en Milieuhygiëne** |
| **CV** | **Curriculum Vitae** |
| **IC** | **Informed Consent** |
| **IMPD** | **Investigational Medicinal Product Dossier** |
| **METC** | **Medical Research Ethics Committee (MREC); in Dutch: Medisch Ethische Toetsingscommissie (METC)** |
| **(S)AE** | **(Serious) Adverse Event** |
| **Wbp** | **Personal Data Protection Act (in Dutch: Wet Bescherming Persoonsgevens)** |
| **WMO** | **Medical Research Involving Human Subjects Act (Wet Medisch-wetenschappelijk Onderzoek met Mensen** |
| **Sponsor** | **The sponsor is the party that commissions the organization or performance of the research, for example a pharmaceutical company, academic hospital, scientific organization or investigator. A party that provides funding for a study but does not commission it is not regarded as the sponsor, but referred to as a subsidizing party.** |
| **DTC** | **Differentiated thyroid carcinoma** |
| **Tg** | **Thyroglobulin** |
| **rhTSH** | **recombinant human Thyrotropin Stimulating Hormone** |
| **US** | **Ultrasound** |
| **mCi** | **milliCurie** |
|  |  |
|  |  |

**SUMMARY**

**Rationale**

Patients with differentiated thyroid cancer (papillary and follicular) are treated with near-total thyroidectomy. In most of the patients this treatment has to be followed by ablation with I-131 to eliminate remnant thyroid tissue to decrease the risk of tumor recurrence and improve sensitivity and specificity of Tg measurement in follow-up. With the introduction of rhTSH the question arises, whether ablative therapy after pretreatment with rhTSH during euthyroidism can be used instead of the classical way of inducing hypothyroidism by withholding suppletion which induces endogenous rise of the TSH level.

**Objectives**

To determine the rate of ablation failure in differentiated thyroid cancer patients using rhTSH instead of thyroid hormone withdrawal for stimulation of remnant tissue, using a fixed dosage of 3.7 GBq (100 mCi).

**Study design**

Prospective multicenter study in the Netherlands.

**Study population**

The maximum number of patients will be 144.

**Intervention (if applicable)**

Two rhTSH injections will be given 6 weeks after total thyroidectomy (before I-131 treatment) and 9 months after the first high dosage I-131treatment.

**Main study parameters/endpoints:**

The primary endpoint of successful ablation is defined as: rhTSH Tg < 1ng/ml, negative rhTSH dxWBS, negative neck US and negative Tg antibodies. If a second (blind) I131 treatment is necessary, according to the Dutch guidelines) the definition of successful ablation is defined as no visible uptake in the original thyroid bed on a post therapeutic scintigraphy.

**Nature and extent of the burden and risks associated with participation, benefit and group relatedness:**

Use of radioactivity means exposure to ionizing radiation. Standard clinical I-131dosage will be given (orally) to patients. Thus, patients will not be exposed to extra radiation. Moreover, I-131 will be cleared more easily when patients are euthyroid instead of hypothyroid, so the radiation burden will decrease.

# INTRODUCTION AND RATIONALE

Patients with differentiated thyroid cancer (papillary and follicular) are treated with near-total thyroidectomy. In most of the patients this treatment has to be followed by ablation with I-131 to eliminate remnant thyroid tissue to decrease the risk of tumor recurrence and improve sensitivity and specificity of Tg measurement in follow-up. With the introduction of rhTSH the question arises, whether ablative therapy after pretreatment with rhTSH during euthyroidism can be used instead of the classical way of inducing hypothyroidism by withholding suppletion which induces endogenous rise of the TSH level.

Several studies have been published about the use of rhTSH preceding I-131 ablation therapy. In two studies a low standard ablation dosage was used after stimulation with rhTSH (30 mCi).^1, 2^ A prospective ablation study in 64 patients from 9 international centers compared the rhTSH pretreatment during thyroxin treatment and thyroxin withdrawal after a high dosage of 100 mCi.^3^ Primary endpoint of the study was the rhTSH stimulated I-131 uptake in the thyroid bed after eight months. The statistical basis of this last mentioned study was based on a non-inferiority framework that was not optimally performed because of a small group size of 21 and 24 patients in both arms. Although the authors reached a different conclusion, this study lacks power and only demonstrates that the two strategies are comparable when a successful ablation rate of 73% is considered acceptable. With this (very liberal) assumption a comparable success of ablation was found with regard to the local thyroid bed. However, this only implicates that an ablation failure rate of 27% is considered ‘non inferior’ to the rate of 7% unsuccessful ablations in the standard endogenous rise of TSH. Recently an update of these patients and no differences have been found in the success of thyroid remnant ablation therapy or clinical outcomes between endogenous TSH stimulation by withholding thyroid hormone therapy vs. exogenous TSH in the euthyroid state in low-risk patients after a median follow-up of 3.7 yr.^4^

A recently published retrospectively rhTSH-assisted remnant ablation had shown similar rates of clinically evident disease recurrence and persistent uptake in the thyroid bed as with the traditional thyroid hormone withdrawal.^5^

Despite these difficulties in the statistical proof of non-inferiority, the potential short-term benefits of rhTSH for the patients with regards to quality of life and socio-economic factors (in medical factors and missed work time) are unequivocal ^6^and make the application of rhTSH attractive. Also whole body radiation is lower after rhTSH stimulation for renal clearance of I-131 is not decreased by hypothyroidism, resulting in a lower total body radiation. A more definitive proof, by prospective studies, that such a strategy is not ultimately harmful to patients, however, is desirable.

The above mentioned discussion has resulted in a recommendation in the Dutch guidelines for the treatment of thyroid cancer that withdrawal of thyroid hormone as well as rhTSH can be used for the ablation therapy (www.oncoline.nl).

Another point of discussion is the optimal activity dosing of radioactive iodine for ablation therapy. In a systematic review of Hackshaw ^7^ various dosage activities of radioactive iodine were compared, by pooling data of observational studies. It was concluded that ablation success was significantly higher by using 100 mCi compared with 30 mCi. However, these findings could not be confirmed in a pooled analysis of randomized controlled trials. A subsequent single-center randomized controlled trial with 72 patients showed that short term rhTSH ablation success were the same (89%) using 50 or 100 mCi radioactive radioiodine.^8^ Although this point needs further clarification, in the currently proposed study we address the question that the ablation using rhTSH pre-treatment is safe in a prospective study.

# OBJECTIVES

**Primary Objective**

To determine whether the failure rate of the I-131 ablation therapy after rhTSH stimulation is equal to the failure rate of I-131 therapy after endogenously stimulated I-131 ablation and using a fixed dosage of 3.7 GBq (100 mCi).

# STUDY DESIGN

Multicenter study in the Netherlands.

A conventional design would be to perform a randomized study comparing TSH preparation with endogenous TSH rise. However, the disadvantage of a randomized study is the tremendous number of patients needed, which is calculated to be 552 patients (based on an acceptable ablation failure rate of 10% versus an alternative hypothesis of 20%, power 90%, alpha 0.05). In addition, even after having performed a randomized study, the main question remains whether the ablation failure rates in the rhTSH arm would be acceptable for widespread application. For example, a statistically higher failure rate after rhTSH could still be considered clinically acceptable.

Therefore, to confirm that the efficacy of rhTSH pre-treatment for ablation therapy is not inferior to withdrawal of thyroid hormone, a fully sequential design will be used, with preset boundaries for stopping in case of inferiority or equality of the rhTSH strategy.

If the failure score by rhTSH passes the upper boundary of the acceptable failure rate then rhTSH is considered inferior to the conventional withdrawal ablation. If failure score after rhTSH passes the lower boundary than rhTSH stimulation is at least equal. This design is less conventional, but is expected to grow significantly in medical research as it provides very clear answers, without the need to confirm what is already known over and over (as is the case in the standard arm of a RCT). ^9^ (See also statistical analysis)

This is a prospective observational study and a fully sequential design will be used, with preset boundaries for stopping in case of inferiority or equality of the rhTSH strategy.

All patients will be treated according protocol using rhTSH (Dutch guidelines). All scan and laboratory evaluation together the treatment decision will be executed in the participating centers. Scans and blood for central reading will be collected and re-evaluated in Groningen every 4 months. This re-evaluation is the study evaluation and will not change the standard treatment.

# STUDY POPULATION

# Population (base)

The maximum number of patients will be 144 (constant alpha=0.014, cumulative alpha=0.05; beta =0.10). This means that with a true failure rate of 10%, the probability of passing the upper boundary is below 5%, with a power of 90%. It is expected that the patients will be recruited within a period of 2 years in the above mentioned centers.

**Inclusion criteria**

- Low and high risk patients (according American Join Committee on Cancer, AJCC 6) with recently diagnosed histological proven DTC, who have to be treated with ablation therapy.

TNM stages T1>1cm, T2, T3, N0, N1, M0 are to be included.

- Aged 18 years or older

- Not pregnant

- No major concurrent diseases leading to a reduced survival of < 1 year

- Normal renal function (serum creatinine level < 130 µmol/l or clearance > 40 ml/min).

**Exclusion criteria**

- Stage T4

- Stage M1 when known before ablation

# Sample size calculation

See Power Analysis, page 14.

# METHODS

**Study procedures (also see flowchart)**

After total thyroidectomy and histological confirmation of differentiated thyroid cancer

substitution therapy is started (levothyroxine) to reach a TSH level < 0.3 mU/l.

- *3-6* weeks post surgery

Neck ultrasound (standard application) to confirm the absence of significant thyroid remnant and to screen for lymph nodes. Basal TSH and Tg measurement.

Additional blood sample for storage and additional blood sample for bone markers

*- 6 weeks post surgery*

0.9 mg rhTSH will be administered i.m. at 0 and 24 hours. (Monday and Tuesday)

At 48 hours 3.7 GBq (100 mCi) I-131 will be administered.

Post treatment scan 7 - 10 days after I-131 application

TSH and Tg measurement at 48 hours. Additional blood sample for bone markers

To ensure the I-deficient diet (according to the Dutch guidelines), the iodine excretion in a 24 hours urine will be measured (in µg/l,) at day 1 (Tuesday).

*- 6 months post ablation therapy*

Neck ultrasound and FNA of suspected nodules (if positive consider re-surgery).

*- 9 months post ablation therapy*

0.9 mg rhTSH will be administered i.m. at 0 and 24 hours (Monday and Tuesday).

At 48 hrs 150 MBq (4 mCi) I-131 application (Wednesday).

TSH and Tg measurement and WBS 48 hours (Friday) after administration of 150 MBq (4 mCi) I-131 (i.e. 96 hours after the first rhTSH). (In case of visible uptake on a planar view, then a SPECT view will be made if available).

Successful ablation is defined when - RhTSH Tg is < 1ng/ml and

- RhTSH dxWBS is negative* and

- Neck US is negative and

- Tg antibodies are negative

* no visible uptake in the thyroid bed or uptake < 0.1% (using a standard gamma probe) on a Dx WBS

In case one or more of the above mentioned diagnostic procedures is positive or in case of a Tg level < 1.0 ng/mL 48 hours at the time of ablation or the possibility of distant metastases, thyroid hormone withdrawal and treatment with 5550 – 7400 MBq (150-200 mCi) I-131 with post-treatment WBS is performed (standard care according to the Dutch guideline). These patients will not be removed from analysis instead the post-treatment scintigrams (as defined earlier) will be evaluated.

Successful ablation (in case of a second (blind) I131 therapy) is defined when -

- Post treatment scintigraphy is negative in the original thyroid bed

In case of a large thyroid remnant or multiple lymph nodes during ultrasound 4-6 weeks post surgery the patients will be excluded from the analysis.

All eligible patients will be treated according to the Dutch national guidelines (See addendum for treatment schedule). The only difference with the standard protocol is the period between the first and second scan. In the protocol it has been delayed to 9 instead of 6 months to eliminate the effect of Tg leakage that occurs for a long period.^10, 11^ There are numerous protocols and guidelines available for the treatment of thyroid cancer. Standard control after ablation therapy has been executed between 6-12 months.^6^ So the period of 9 months that has been chosen in this protocol is in accordance with the standard protocols that are available for thyroid cancer treatment.

*Thyroglobulin assay*

For the re-evaluations at the University Medical Centre Groningen Tg concentrations are measured using a commercial immunoradiometric assay (Brahms Tg-Plus, Heningsdorf, Germany) in a coated tube system. The Brahms Tg-Plus assay is claimed to be calibrated against the CRM 457 standard and is correlated with the former Nichols Advantage Tg assay ^12^ according to the following formula: Brahms assay = 0.3 x Nichols assay. In addition, the Brahms assay has an analytical sensitivity of 0.1 ng/ml and a functional sensitivity of 0.3 ng/ml (personal findings). Tg antibodies will also be measured by Brahms assay (<46 U/ml)

*Storage of body material*

It becomes clear more and more that growth factors in the blood are up regulated in the

presence of malignancy. To examine new promising tumor markers in the future, blood will be collected by vena punction with a maximum of 50 ml and stored.

Storage of material from this relatively large patient group with DTC

Is of great interest taking into account the rarity of these inherited diseases. Additionally, the current little understanding of the pathogenesis of thyroid cancer makes storage desirable, since new research may be conducted that will give more insight into the pathogenesis.

All human body material will be stored in a freezer with a temperature of -80˚C for a period of 10 years. For storage patient’s approval will be asked explicitly as a part of the informed consent. To determine whether the levels of in the future defined markers are predictors of disease activity or disease progression, clinical data will be available in order to identify important correlations. All body samples will be stored anonymously, however the study doctor is capable to trace back the patients. This is necessary in case of findings that will give additional insight in patient’s health.

*Main study parameter/endpoint*

The primary endpoint of successful ablation is defined above. Uptake will be quantified by defined methods using standard gammaprobe. The scans will be evaluated both by local and central reading:

Measurement of uptake :

A standard gammaprobe, calibrated for I-131, and similar to the one used for measuring thyroid uptake in hyperthyroidism, will be used. Measurements will be performed as described in the Dutch Recommendations for Nuclear Medicine procedures.

Central reading facility: after local reading, every 4 months post-therapy scans as obtained in the study centre will be forwarded to the central site, preferably in DICOM format to allow modification of image intensity to allow central interpretation. In case of differences in reading a consensus will be reached by the involved interpreters. Probe measurements will not be centralized.

**Withdrawal of individual subjects**

Subjects can leave the study at any time for any reason if they wish to do so without any consequences. The investigator can decide to withdraw a subject from the study for urgent medical reasons.

## Replacement of individual subjects after withdrawal

In case of individual withdrawal, statistical analysis must determine if replacement is necessary for the study results. If this is the case, other individuals will be included in the study as a new subject.

## Premature termination of the study (see Statistical Analysis, page 14)

During the study every 3 months evaluation will take place of scans and laboratory measurements. When the lower boundary is passed, sequential monitoring can be stopped, interim statistical analysis will be done and the use of rhTSH will become the standard treatment.

Final statistical analysis will be done after completion of all 144 patients.

When the upper boundary is passed, the study will be stopped prematurely and the use of rhTSH (with dosage and timing as in the protocol) can not become the standard treatment. Final statistics will be provided.

## SAFETY REPORTING

## Section 10 WMO event

In accordance to section 10, subsection 1, of the WMO, the investigator will inform the subjects and the reviewing accredited METC if anything occurs, on the basis of which it appears that the disadvantages of participation may be significantly greater than was foreseen in the research proposal. The study will be suspended pending further review by the accredited METC, except insofar as suspension would jeopardize the subjects’ health. The investigator will take care that all subjects are kept informed.

## Adverse and serious adverse events

No adverse or serious events are to be expected when using rhTSH or during scanning. In previous studies using rhTSH no adverse effects have been reported.

Adverse events are defined as any undesirable experience occurring to a subject during a clinical trial. All adverse events reported spontaneously by the subject or observed by the investiga­tor or his staff will be recorded.

A serious adverse event is any untoward medical occurrence or effect that at any dosage results in death;

- is life threatening (at the time of the event);
- requires hospitalization or prolongation of existing inpatients’ hospitalization;
- results in persistent or significant disability or incapacity;
- is a congenital anomaly or birth defect;
- is a new event of the trial likely to affect the safety of the subjects, such as an unexpected outcome of an adverse reaction, lack of efficacy of an IMP used for the treatment of a life threatening disease, major safety finding from a newly completed animal study, etc.

All SAEs will be reported to the accredited METC that approved the protocol, according to the requirements of that METC.

##

## *Follow-up of adverse events*

All AEs will be followed until they have abated, or until a stable situation has been reached. Depending on the event, follow up may require additional tests or medical procedures as indicated, and/or referral to the general physician or a medical specialist.

#

# STATISTICAL ANALYSIS

# Descriptive statistics

**Power analysis**

Data on ablation rates.

Based on literature and local Dutch data the investigators agree that a mean ablation failure rate up to 10% is considered acceptable.

Definition of ablation success : A recent review has analyzed the success rate of ablation at 80% with 100mCurie after withdrawal.^7^ Results from Dutch centers vary from 88% , 93% and lower (68% and 86%) and 56%.^13-15^

A historical ablation success rate defined on the basis of the post therapeutic scan in those patient with a second blind I131 therapy will follow.

Event = ablation failure as defined above

Event =

ablation failure as defined above

Figure 1

*Background of the sequential design with stopping rule*

A sequential design with a stopping rule equipped with two boundaries, gives the opportunity to use every single consecutive ablative therapy in a daily executed interim analysis.(see fig.1)

The preset upper boundary allows premature stopping in case of an observed failure rate that is inferior to the acceptable rate of 10%, with a rate of 20% as alternative ( Total maximal number of patients 144; constant alpha=0.014, cumulative alpha=0.05; beta =0.10). This means that with a true failure rate of 10%, the probability of passing the upper boundary is below 5%, with a power of 90%.

The preset lower boundary allows premature stopping for futile testing (constant alpha=0.2638, cumulative beta=0.10; cumulative alpha=0.05). This means that with a true failure rate of 20%, the probability of passing the lower boundary is below 10% and that the probability of subsequent passage of the upper boundary would be below 5%.

The 50% probability of stopping (because of passing the upper or lower boundary) in case of a 20% failure rate is reached within the first 50 consecutive patients, whereas the 50% probability of stopping in case of a 10% failure rate is reached within 77 patients.

During the study, there is an analysis after entering each new patient. When the lower boundary is passed, sequential monitoring can be stopped, interim statistical analysis will be done and the use of rhTSH will become the standard treatment. Final statistical analysis will be done after completion of all 144 patients.

When the upper boundary is passed, the study will be stopped prematurely and the use of rhTSH (with dosage and timing as in the protocol) can not become the standard treatment. Final statistics will be provided. Then two alternatives can be followed: withdrawal remains the standard treatment or a new study is started with different time and/or dosing of rhTSH.

The probabilities of stopping with various true failure rates are given in figure 2.

Figure 2 Probability of stopping at different failure rates 5 -25%

*Stopping rule with two boundaries*

*The upper boundary*

For one group of patients we can investigate inferiority of the failure rate p to H0: p<=p0 with the alternative H1:p>=p1. With a fully sequential design an upper boundary UB(n) can be constructed, being the maximally accepted number of events within n consecutive patients.

If the failure rate is p, then P(p,n-1,UB(n-1)) is the probability that the number of failures after (n-1) patients is UB(n-1). When the chosen one-sided level of significance is alpha(n-1), then P(p,n-1,Ub(n-1))>= alpha(n-1). Also alpha(n) has been chosen. In case an event occurs in patient n, there are two possibilities:

1) (P(p,n-1,UB(n-1)))*p=P(p,n,UB(n-1)+1)<alpha(n). This would mean that this result

is unacceptable, activating the stopping rule. Thus the upper boundary has been

passed, making UB(n-1)=UB(n). The probability of passage is P(p,n,UB(n)+1).

2) (P(p,n-1,UB(n-1)))*p=P(p,n,UB(n-1)+1)>=alpha(n). This would mean that this

result is acceptable, leaving the stopping rule inactivated. The upper boundary is

not passed, making UB(n)=UB(n-1)+1. The probability of passage is

P(p,n,UB(n)+1)=0.

The cumulative probability P(sum UB) to pass the upper boundary is the sum of P(p,n,UB(n)+1) from n=1 to n=N. N is the maximum of patients needed.

For p=p0 P(p0,sum UB) should not exceed a chosen value of alpha(rule). And for p=p1 P(p1,sum UB) should exceed a chosen value of (1-beta(rule)), being the power.

In this way the upper boundary serves as a fully sequential test of inferiority. When the boundary is passed, we do not accept H0 and declare the failure rate inferior to p0.

*The value of alpha(n).*

1. With alpha(n) declining with rising n, as with the method of Pocock ^16^, the probability of "discordant" activation of the stopping rule is larger than with a constant alpha(n).^17^ Following the method of O'Brien en Fleming with a constant alpha(n)^18^, one would expect alpha(n)=alpha(rule)/N. This is not the case, because UB(n) is discrete in nature. Alpha(n) is larger than alpha(rule)/N. Z(1-alpha(n)) approximates Z(1-alpha(rule) +CTC. CTC is the socalled Christmas tree correction, which is approximatedly 0.583 in size.^19^

*The calculation of UB(n)*

Conventionally, UB(n) approximates n*p0+Z(1-alpha(n))*(n*p0)^0.5. Because of the discrete character of UB(n) this transforms into:

UB(n) = integer{n*p0 +Z(1-alpha(n)) *(n*p0)^0.5 +1}.

*The value of N*

The value of N can now be calculated as

N= integer{[Z(1-alpha(n)) *(p0)^0.5+Z(1-beta(N))*(p1)^0.5/(p1-p0)]^2+1}.

*The lower boundary*

*Futile testing*.

In a sequential design a stopping rule can be equipped with a lower boundary LB(n). Passage of that boundary would mean futile testing and monitoring could be stopped, accepting H0.

When constructed in such a way that UB(N)=LB(N), we know that the probability to pass the lower boundary would be 1-alpha(rule) in case p=p0 and (1-beta(rule)) in case p=p1, because of the limits set for UB(n).

Suppose the number of events after (n-1) patients equals LB(n-1) and LB(n)=LB(n-1)+1. If the failure rate is p1 and no events occur in patient n, then P(p1,n,LB(n))-1 is the probability that the number of failures after n patients is just 1 below LB(n). The overall probability to subsequently pass the upper boundary within the next (N-n) patients would be P(futile,n). P(futile,n)=0 in case LB(n)=LB(n-1). The sum P(p1, sum LB) of these probabilities P(futile,n) summed from n=1 to n=N we call alpha(futile). Alpha(futile) must be smaller than beta(rule).

Such a lower boundary can be approximated by:

LB(n)= integer{UB(N)+1-p1*(N-n)-Z(1-alpha1(n))*(p1*(N-n))^0.5)>=0. We choose alpha1(n) to be constant and with such a value that alpha(futile) is the result.

*Simultaneously active boundaries.*

The boundaries given above are still approximations. With low values of p0 and p1 the approximations do deviate from exact calculations. For this reason alone all probabilities should be calculated exactly. But there is a second reason to do so: the probability to pass one boundary is influenced by the probability to pass the other one.

Such exact calculation can be done in a spread sheet programme. The calculations are repeated with changing values of the Z-parameters until the values of alpha(rule), (1-beta(rule)) and alpha(futile) meet the limits set.

Estimation of the failure rate p after activation of the stopping rule.

If a boundary is passed after k patients, with a total number of events of (UB(n)+1) for the upper boundary or of( LB(n)-1) for the lower boundary, p can be estimated.

When the true failure rate is p, then

a) After passing the upper boundary: the sum of (P(p,n,UB(n)+1) from n=1 to n=k is

0.5 for the estimation of p, whereas this sum is alpha(rule) for the upper limit of its

100*(1- 2*alpha(rule)) % confidence interval (CI) and 0.95 for the lower limit of this

CI.

b) After passing the lower boundary: the sum of (P(p,n,LB(n)-1) from n=1 to n=k is

0.5 for the estimation of p, whereas this sum is alpha(rule) for the lower limit of its

100*(1- 2*alpha(rule)) % confidence interval (CI) and 0.95 for the upper limit of this

CI.

# ETHICAL CONSIDERATIONS

## Regulation statement

This study will be conducted according to the principles of the Declaration of Helsinki (latest version, 2008, Seoul, www.wma.net) and in accordance with the Medical Research Involving Human Subjects Act (WMO).

Use of radioactivity means exposure to ionizing radiation. Because of the potential hazards of radiation, guidelines for the exposure of healthy volun­teers are specified in “Besluit Stralingsbescherming (BS 2000), artikel 60, Staatsblad 2001, 397", according to the guidelines of the International Commission on Radiological Protection (ICRP).

Standard clinical 131I dosage will be given (orally) to patients. Thus, patients will not be exposed to extra radiation.

## Recruitment and consent

Patients will first be informed orally about the purpose by their treating doctors. Thereafter patients will also be informed about this study by the study coordinator of each center, and will have to give their written informed consent (in accordance with the Declaration of Helsinki and the Dutch legal regulation (Wet Medisch Wetenschappelijk Onderzoek met Mensen) in order to participate in the study. After receiving the written information patients will have two weeks time for reflection. Both orally and in writing it will be emphasized that participation in the study is by patient’s own volition. Patients’ withdrawal at any time from the study will neither have consequences for their most optimal treatment nor will it affect their relation to their treating endocrinologist. Dr. J. Pruim(independent medical doctor) University Medical Center Groningen, phone (050)-3612205­, is not involved in this study and can give further information regarding this study.

## Benefits and risks assessment, group relatedness

The study population will not include minors and/or incapacitated adults. The study coordinator of each center will explain subjects that they will not benefit in any particular way from participation in the study.

## Compensation for injury

The sponsor/investigator has a liability insurance which is in accordance with article 7, subsection 6 of the WMO.

The sponsor (also) has an insurance which is in accordance with the legal requirements in the Netherlands (Article 7 WMO and the Measure regarding Compulsory Insurance for Clinical Research in Humans of 23th June 2003). This insurance provides cover for damage to research subjects through injury or death caused by the study.

1. € 450.000,-- (i.e. four hundred and fifty thousand Euro) for death or injury for each subject who participates in the Research;
2. € 3.500.000,-- (i.e. three million five hundred thousand Euro) for death or injury for all subjects who participate in the Research;
3. € 5.000.000,-- (i.e. five million Euro) for the total damage incurred by the organisation for all damage disclosed by scientific research for the Sponsor as ‘verrichter’ in the meaning of said Act in each year of insurance coverage.

The insurance applies to the damage that becomes apparent during the study or within 4 years after the end of the study.

## Incentives (if applicable)

Patients will be refunded for their travel expenses.

# ADMINISTRATIVE ASPECTS AND PUBLICATION

## Handling and storage of data and documents

The information will be handled carefully and privately. As for patient care the data will be kept.

To guarantee anonymity codes will be used for the research data. All participating hospitals will have their specific codenumber after which a patientnumber and the first letter of the patient’s surname (for women their maiden name) will follow.

Serum samples collected for Tg measurement are stored to explore relationships between these tumormarkers and changes in levels of VEGF pathway related biomarkers and endothelial activation markers.

## Amendments

Amendments are changes made to the research after a favourable opinion by the accredited METC has been given. All amendments will be notified to the METC that gave a favourable opinion.

## Annual progress report

The sponsor/investigator will submit a summary of the progress of the trial to the accredited METC once a year. Information will be provided on the date of inclusion of the first subject, numbers of subjects included and numbers of subjects that have completed the trial, serious adverse events/ serious adverse reactions, other problems, and amendments.

## End of study report

The investigator will notify the accredited METC of the end of the study within a period of 8 weeks. The end of the study is defined as the last patient’s last visit.

In case the study is ended prematurely, the investigator will notify the accredited METC, including the reasons for the premature termination.
Within one year after the end of the study, the investigator will submit a final study report with the results of the study, including any publications/abstracts of the study, to the accredited METC.

## Public disclosure and publication policy

With the obtained research data a scientific article will be created. This article will be submitted in a peer reviewed journal. Full disclosure will respect the privacy of the subject.

Reference List

1. Barbaro D, Boni G, Meucci G et al. Radioiodine treatment with 30 mCi after recombinant human thyrotropin stimulation in thyroid cancer: effectiveness for postsurgical remnants ablation and possible role of iodine content in L-thyroxine in the outcome of ablation. J Clin Endocrinol Metab 2003; 88(9):4110-4115.

2. Pacini F, Molinaro E, Castagna MG et al. Ablation of thyroid residues with 30 mCi (131)I: a comparison in thyroid cancer patients prepared with recombinant human TSH or thyroid hormone withdrawal. J Clin Endocrinol Metab 2002; 87(9):4063-4068.

3. Pacini F, Ladenson PW, Schlumberger M et al. Radioiodine ablation of thyroid remnants after preparation with recombinant human thyrotropin in differentiated thyroid carcinoma: results of an international, randomized, controlled study. J Clin Endocrinol Metab 2006; 91(3):926-932.

4. Elisei R, Schlumberger M, Driedger A et al. Follow-up of low-risk differentiated thyroid cancer patients who underwent radioiodine ablation of postsurgical thyroid remnants after either recombinant human thyrotropin or thyroid hormone withdrawal. J Clin Endocrinol Metab 2009; 94(11):4171-4179.

5. Tuttle RM, Brokhin M, Omry G et al. Recombinant human TSH-assisted radioactive iodine remnant ablation achieves short-term clinical recurrence rates similar to those of traditional thyroid hormone withdrawal. J Nucl Med 2008; 49(5):764-770.

6. Luster M, Felbinger R, Dietlein M, Reiners C. Thyroid hormone withdrawal in patients with differentiated thyroid carcinoma: a one hundred thirty-patient pilot survey on consequences of hypothyroidism and a pharmacoeconomic comparison to recombinant thyrotropin administration. Thyroid 2005; 15(10):1147-1155.

7. Hackshaw A, Harmer C, Mallick U, Haq M, Franklyn JA. 131I activity for remnant ablation in patients with differentiated thyroid cancer: A systematic review. J Clin Endocrinol Metab 2007; 92(1):28-38.

8. Pilli T, Brianzoni E, Capoccetti F et al. A comparison of 1850 (50 mCi) and 3700 MBq (100 mCi) 131-iodine administered doses for recombinant thyrotropin-stimulated postoperative thyroid remnant ablation in differentiated thyroid cancer. J Clin Endocrinol Metab 2007; 92(9):3542-3546.

9. van der Zee AG, Oonk MH, De Hullu JA et al. Sentinel node dissection is safe in the treatment of early-stage vulvar cancer. J Clin Oncol 2008; 26(6):884-889.

10. Baudin E, Do Cao C, Cailleux AF, Leboulleux S, Travagli JP, Schlumberger M. Positive predictive value of serum thyroglobulin levels, measured during the first year of follow-up after thyroid hormone withdrawal, in thyroid cancer patients. J Clin Endocrinol Metab 2003; 88(3):1107-1111.

11. Torlontano M, Attard M, Crocetti U et al. Follow-up of low risk patients with papillary thyroid cancer: role of neck ultrasonography in detecting lymph node metastases. J Clin Endocrinol Metab 2004; 89(7):3402-3407.

12. Persoon AC, Links TP, Wilde J, Sluiter WJ, Wolffenbuttel BH, Van Den Ouweland JM. Thyroglobulin (Tg) recovery testing with quantitative Tg antibody measurement for determining interference in serum Tg assays in differentiated thyroid carcinoma. Clin Chem 2006; 52(6):1196-1199.

13. de Klerk JM, de Keizer B, Zelissen PM, Lips CM, Koppeschaar HP. Fixed dosage of 131I for remnant ablation in patients with differentiated thyroid carcinoma without pre-ablative diagnostic 131I scintigraphy. Nucl Med Commun 2000; 21(6):529-532.

14. Links TP, van Tol KM, Jager PL et al. Life expectancy in differentiated thyroid cancer: a novel approach to survival analysis. Endocr Relat Cancer 2005; 12(2):273-280.

15. Verkooijen RB, Verburg FA, van Isselt JW, Lips CJ, Smit JW, Stokkel MP. The success rate of I-131 ablation in differentiated thyroid cancer: comparison of uptake-related and fixed-dose strategies. Eur J Endocrinol 2008; 159(3):301-307.

16. Pocock SJ. Group sequential methods in the design and analysis of clinical trials, Biometrika 1977,64,191-199. 2010.
Ref Type: Generic

17. Choi CaLY. Interim analyses with delayed observations in clinical Trials, Statist.Med, 1999,18,1297-1306. 2010.
Ref Type: Generic

18. O'Brien PaFT. A multiple testing procedure for clinical trials; Biometrics 1979,35,549-556. 2010.
Ref Type: Generic

19. Whitehead J. The Design and Analysis of Sequential Clinical Trials; 1991, 2nd edn, Ellis Horwood, Chichester. 2010.
Ref Type: Generic
